# Molecular Epidemiology, Drug Susceptibility and Economic Aspects of Tuberculosis in Mubende District, Uganda

**DOI:** 10.1371/journal.pone.0064745

**Published:** 2013-05-31

**Authors:** Adrian Muwonge, Sydney Malama, Tone B. Johansen, Clovice Kankya, Demelash Biffa, Willy Ssengooba, Jacques Godfroid, Berit Djønne, Eystein Skjerve

**Affiliations:** 1 Department of Food Safety and Infection Biology, Centre for Epidemiology and Biostatistics, Norwegian School of Veterinary Science, Oslo, Norway; 2 Department of Health Sciences, Evelyn Hone College, Lusaka, Zambia; 3 Norwegian Veterinary Institute, Oslo, Norway; 4 Department of Public Health and Preventive Medicine, College of Veterinary Medicine, Animal Resources & Biosecurity, Makerere University, Kampala, Uganda; 5 College of Medicine, University of Arizona, Tucson, Arizona, United States of America; 6 Mycobacteriology Laboratory, Department of Medical Microbiology, Makerere University College of Health Sciences, Kampala, Uganda; 7 Department of Food Safety and Infection Biology, Section of Arctic Veterinary Medicine, Norwegian School of Veterinary Science, Tromsø, Norway; 8 The Roslin Institute, University of Edinburgh, Easter Bush Roslin, Midlothian, United Kingdom; St. Petersburg Pasteur Institute, Russian Federation

## Abstract

**Background:**

Tuberculosis (TB) remains a global public health problem whose effects have major impact in developing countries like Uganda. This study aimed at investigating genotypic characteristics and drug resistance profiles of *Mycobacterium tuberculosis* isolated from suspected TB patients. Furthermore, risk factors and economic burdens that could affect the current control strategies were studied.

**Methods:**

TB suspected patients were examined in a cross-sectional study at the Mubende regional referral hospital between February and July 2011. A questionnaire was administered to each patient to obtain information associated with TB prevalence. Isolates of *M. tuberculosis* recovered during sampling were examined for drug resistance to first line anti-TB drugs using the BACTEC-MGIT960^TM^system. All isolates were further characterized using deletion analysis, spoligotyping and MIRU-VNTR analysis. Data were analyzed using different software; MIRU-VNTR *plus*, SITVITWEB, BioNumerics and multivariable regression models.

**Results:**

*M. tuberculosis* was isolated from 74 out of 344 patients, 48 of these were co-infected with HIV. Results from the questionnaire showed that previously treated TB, co-infection with HIV, cigarette smoking, and overcrowding were risk factors associated with TB, while high medical related transport bills were identified as an economic burden. Out of the 67 isolates that gave interpretable results, 23 different spoligopatterns were detected, nine of which were novel patterns. T2 with the sub types Uganda-I and Uganda-II was the most predominant lineage detected. Antibiotic resistance was detected in 19% and multidrug resistance was detected in 3% of the isolates.

**Conclusion:**

The study detected *M. tuberculosis* from 21% of examined TB patients, 62% of whom were also HIV positive. There is a heterogeneous pool of genotypes that circulate in this area, with the T2 lineage being the most predominant. High medical related transport bills and drug resistance could undermine the usefulness of the current TB strategic interventions.

## Introduction

Tuberculosis (TB) caused by *Mycobacterium tuberculosis* remains a major global public health problem regardless of the modern diagnostics, chemotherapy and vaccines [1] [2]. The WHO estimates that more than one third of the world’s population has been exposed to *M. tuberculosis*, of which approximately 10% will develop active TB during their lifetime [3]. In 2011 alone, there were 8.7 million new TB cases reported, of which 1.4 million patients succumbed to the disease [3]. Case fatality rates among HIV infected patients are extremely high, and can be up to 40% in sub Saharan Africa [4], [5]. Uganda is ranked 16^th^ among the 22 countries with the highest TB burden, with an incidence of 299 and a mortality rate of 84 cases per 100,000 per year [6]. TB is endemic in poor urban and peri-urban areas mainly due to congested living conditions, high HIV prevalence and malnutrition [7]. Lifestyles have recently become the center of focus with regards to activation and mortality of TB. Studies carried out in Taiwan and Nigeria showed that active TB was predominant among smokers resulting in more case fatalities as compared to nonsmokers [8], [9]. In rural Uganda, TB transmission has been associated with sharing of cigarettes and straws used in local brew consumption [10].

The global TB outlook is grim due to the development of multi drug resistant strains (MDR-TB) which do not respond to the first line TB therapy (rifampicin and isoniazid) [11], [12]. MDR-TB patients require longer, more expensive and more toxic treatment regimens, and yet they are less likely to be cured. This represents an enormous strain on the control of TB in endemic countries with poor resources, like Uganda [13], [14]. According to the latest WHO reports, MDR-TB prevalence among new and previously treated patients in Kampala is 1.1% and 11.7%, respectively [12], remarkably higher than the national MDR-TB prevalence detected in 1999 [14]. The true picture of MDR-TB in HIV hotspots like the Mubende district is however yet to be accurately established. Until then, surveillance of resistance to TB therapeutics and evaluation of the effectiveness of direct observed treatment short course (DOTS) at a national level remains the cornerstone for any TB control program in Uganda [15].

Tuberculosis is often referred to as a poverty driven disease because malnutrition and other diseases predispose latently infected individuals to TB activation, while poor hygiene practices and close contact between people enhances transmission [16], [17]. The disease also maintains poverty as it affects the most productive sub population of the society, the young adults [16]–[18]. It is reported that even when other national characteristics are taken into account, countries with a lower TB burden grow faster than those heavily afflicted [16]–[18]. Therefore, the link between ill health and impoverishment has placed health matters high on the agenda of most developing countries. The problem seems to arise from the inequitable distribution of quality health care services which then creates distance and cost barriers for poor communities [16], [18]. Women are the most affected in these areas because of the triple burden of housework, childcare and employment. This allows them very little time to access health centers for TB treatment [18], [19]. And, as TB is a chronic condition that requires regular treatment and special nutrients for nourishing the sick, costs over time are inevitable.

Molecular epidemiology is a suitable surveillance tool for this old but fast evolving epidemic as molecular markers are used to determine whether isolates of the same mycobacterial species recovered from either the same or different sources are possibly related [20], [21]. This gives the possibility to describe geographical transmission and trends of strains or clones and to identify risk factors for infection, which eventually will help to develop measures of breaking transmission chains [22]. Earlier studies based on biochemical tests showed that 67% and 32% of patients in Mulago referral hospital in Kampala, Uganda were infected with *M. africanum* subtype II and *M. tuberculosis*, respectively [23]. However, with the advent of PCR based molecular typing like spoligotyping, these findings have been refined. The common feature in the spoligotype pattern for most isolates from Kampala was the lack of spacer 33–36, 40 and 43. At the time, these profiles were assigned to *M. africanum* sub types Uganda-I and Uganda-II [24], [25]. Today, these genotypes are known to be variants of *M. tuberculosis*, but still the names Uganda-I and II are commonly used [25], [26]. Recently, MIRU-VNTR has been used to investigate clonal complexity and distribution of *M. tuberculosis* genotypes in clinical TB in central and south western Uganda [27], [28]. Combining spoligotyping and MIRU-VNTR is reported to give a competitive edge over exclusively using one method [21], [29].

Mubende is the poorest district in the central region of Uganda with an HIV prevalence of 18% [15], creating an immune compromised sub population susceptible to TB infections. To date there is no study conducted to elucidate the dynamics of circulating TB genotypes in this area. This study therefore aimed at investigating genotypic characteristics and drug resistance profiles of *M. tuberculosis* isolates from suspected TB patients. Furthermore, risk factors and economic burdens that could affect the current control strategies were studied.

## Materials and Methods

### Ethical Considerations

Full ethical clearance was obtained from the Uganda National Council for Science and Technology (UNCST). Healthcare authorities and the research team were briefed about the ethical issues. Oral consent was obtained from participating patients, next of kin or caretakers of minors instead of written consent [30]. All the adjustments were approved as per research ethical mandate given UNCST. Furthermore, data was anonymously analyzed as stipulated by the UNCST guidelines of research involving human as research participants (2.2/b-e/2007).

### Study Site

Mubende district is located in the central region of Uganda, administratively divided into two counties namely; Buwekula and Kassanda. The counties are further sub-divided into ten sub-counties; Bagezza, Butologo, Kasambya, Kitenga, Kiyuni, Madudu, Bukuya, Kassanda, Kiganda and Myanzi. Mubende is inhabited by approximately 750,000 people, of which 64% live below the poverty line in population dense urban and peri-urban sub counties of Bagezza specifically Mubende town council [30] [15]. Mubende is indeed a convergence of culture and ethnicity with the majority of inhabitants belonging to the Bantu ethnic group (Ganda, Basoga, Nyoro, Batooro, Banyankole, Bafumbira), Nilotics (Acholi) and Hamite (Nyarwanda) ethinic groups [15]. Mubende has one of the lowest TB detection levels in Uganda and the district’s national TB program is severely hit with treatment adherence problems [31].

### Study Design and Population

This cross sectional study was conducted between February and July 2011 with the following inclusion criteria; the patient had to have presented with cervical lymphadenitis and/or a cough that had persisted for at least two weeks at the Mubende regional referral hospital. The national tuberculosis program (NTP) is only run at this referral hospital in the Mubende district and all persistent ailments are treated here. This implies that the majority of the residents in this district who met the study’s criteria would most likely be treated at this facility. The samples collected over the six months period would therefore give a cross sectional window into the population dynamics of the district. A total of 344 patients met the above criterion; 41 at the outpatient department (OPD), 216 at the tuberculosis ward, eight at the pediatric ward and 79 at the HIV/AIDS department run by the SUSTAIN program under the Joint clinical research council (JCRC). Part of the material obtained from this study, focusing on multiple strain infections, has been published elsewhere [30].

In addition to sample collection, bio data (sex, age, marital status, weight height etc.) and clinical history such as HIV status was collected. A questionnaire was administered by an experienced nursing officer to obtain information about the geographical, socioeconomic and other factors associated with TB prevalence. The economic analysis was based on income and expenditure questions included in Questionnaire S1 [18], [19]. To minimize the level of bias, patients were orientated about the objectives of this study. Furthermore, the questions were asked or paraphrased in local languages without changing the gist of the question. The analysis was done with an intention of giving an insight into incomes as well as medical related spending behavioral patterns among TB patients.

### Sample Collection, Culturing and Identification of Mycobacteria

Sputum and/or lymph node aspirates were collected by qualified and experienced medical personnel. One sample was collected per patient who had either a cough or lymphadenitis, and two from those who had both clinical presentations (one from each anatomical compartment). The samples were analyzed at the bio-safety level three (BSL3) tuberculosis laboratory at Makerere University College of Health sciences, and subjected to culture using standard operating procedures [31], [32] [30], [32], [33]. Cultures were incubated for up to eight weeks on Lowenstein Jensen (LJ) slants (BD BBL™; Franklin lakes, NJ, USA) and six weeks in *Mycobacterium* Growth Indicator Tube (MGIT960) (BD BBL™) according to the manufactures’ manual. Smears were made from each sample and stained for fluorescent microscopy. Results were communicated to the patients’ health facilities for routine management. Cultures positive for mycobacteria were subjected to Capilia Neo™ assay (TAUN, Numazu, Japan) to differentiate between the *M. tuberculosis* complex (MTC) and non tuberculous mycobacteria [34]. Results were interpreted as described by Muwonge et al [30]. Secondary pure cultures were then forwarded for DNA harvesting. Results were entered into a computerized laboratory access database and linked with individual patient records on the sample reporting form.

### Drug Resistance Assay

The MTC cultures were subjected to drug susceptibility testing using the BACTEC MGIT960™ (BD BBL™) automated system for detection of mycobacterial growth and drug susceptibility according to the manufactures’ manual [33]. Due to logistical reasons, only the four most used first line TB drugs in Mubende district were evaluated; isoniazid, rifampicin, ethambutol and streptomycin [30]. The drug susceptibility test was only run on the 67 isolates which gave results on molecular typing.

### Molecular Analysis

Genomic DNA was extracted by heat inactivation of culture material dissolved in TE buffer and direct use of the supernatant as template [30]. The standard PCR amplification of the genomic regions of difference (deletion analysis) was performed as previously described [34] at the Norwegian Veterinary Institute, Oslo. A set of primers including RD1, RD4, RD9 and RD12 were used, differentiating the members of the *M. tuberculosis* complex.

Spoligotyping were done by Genoscreen® (Lille, France), based on the protocol using the primers DRa and DRb as described by [35], [36]. Chromosomal DNA of *M. tuberculosis* strain H37Rv and *M. bovis* BCG were used as positive controls and water as a negative control.

MIRU-VNTR with the standardized panel of 15 loci (424, 577, 580, 802 960 1644 1955, 2163b, 2165, 2401, 2996, 3192, 3690, 4052, 4156) was performed at Genoscreen® [36] using triplex PCR with fluorescent primers. DNA fragments were separated by capillary electrophoresis and the size of the PCR products and allel assignment was determined with GeneMapper® Software (Applied Biosystems, Paris, France). The results were reported in Roman numerals representing the number of repeats per locus. The standard 15 locus MIRU-VNTR was used because it has been documented to sufficiently define phylogeny and evolution of *M. tuberculosis* complex with minimum resource input [37]. Given that this study was conducted in and for a resource limited country, the 15 locus MIRU-VNTR was regarded as sufficient for analysis.

### Data Assembly and Analysis

Information obtained from the questionnaires, HIV status from case history and corresponding culture results for each patient were entered and validated in Excel® 2007. The data were then imported into Stata (Stata ver. 11/SE for windows, Stata Corp, College station, TX) for statistical analyses. A univariable association between *M. tuberculosis* presence or absence was computed with cut off set at p≤0.25. Variables that met the above criterion were then included in both the mixed logistic and linear regression models for risk and economic factors associated with TB respectively, with sub-county as the random effect part of the logistic model and tribe for the linear regression. Standard methods were used to assess the model fit and validity. Molecular results from Genoscreen® and the Norwegian Veterinary Institute were entered and validated in Excel® 2007. These were then copied into MIRU-VNTR*plus* [38], SITVITWEB http://www.pasteur-guadeloupe.fr:8081/SITVIT_ONLINE/ [39] and BioNumerics® in order to establish the lineage, sub-types and spoligotype international types (SIT) designations and generate dendograms. Calculation of discriminatory power was done as described by Hunter and Gaston [40].

To analyze the genetic relationship and possible mutation pathways within the dominant clusters, a minimum spanning tree (MST) based on a single locus variation (SLV) on MIRU-VNTR was generated using BioNumerics® version 6.1 (Applied Maths, Sint-Martens-Latem, Belgium). MST is a tool that takes a unidirectional graph and extracts the sub-graphs with the smallest weights [41]. In this study, the MST was created in a number of steps. First, a cluster analysis was performed on all strains using the spoligotypes to identify the dominant clusters. Based on the results from the cluster analysis a unidirectional network was created based on the MIRU-VNTR results of the predominant clusters. The nodes (circles) consist of identical genotypes and the edges (lines) of weights based on number of mutations (steps) taken from the loci used [41]. Long weights (steps) indicate multiple mutations, while short weights indicate few mutations. The MST algorithm was then applied to this graph to extract all sub-graphs with the minimal overall weight sum. Hence, the most similar strains are clustered closely together with short and thick edges, while increasing genomic variation leads to thin and longer edges. The genetic relatedness was further expressed as a percentage based on the formula below.
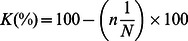



*K* is the genetic relatedness and n is the number of steps between any two clonal variants. A step means the number of SLV at which any two clonal variants differ, and is indicated as numbers from 1–7 on the MST. Therefore each step represents a difference at a single locus variation. N is the total number of loci used in the MIRU-VNTR analysis.

## Results

### Summary Statistics of Patients

Out of the 344 studied patients, 206 (60%) were male and 138 (40%) were female, with an average age of 31.5 (30.2–33.0) years, height of 155.7 (153.8–157.4) cm and weight of 50.4 (49.2–51.55) kg. Forty-seven percent of the patients were married and 41% single, the rest widowed, divorced or too young to be categorized. Sputum and cervical lymph node aspirates accounted for 85% and 15% of the total clinical samples collected respectively. The records on HIV status revealed that 85 (24.7%) were HIV positive, 59 (17%) negative and 203 (59%) had unknown status.

Of the 344 patients, 61 were smear positive on direct fluorescent microscopy, while *M. tuberculosis* was isolated from 74 (21.5%) cases. Of the culture positive patients, 51 (68%) were newly diagnosed and 23 (32%) were previously treated for TB infections. Pulmonary TB was the predominant disease, manifested in 88% of the sampled group. HIV/TB co- infections were detected in 48 (65%) of the culture positive patients. The TB prevalence was highest and lowest among the Bafumbira (33%) and Basoga (7%) ethnic groups, respectively (Table 1).

**Table 1:** Prevalence of tuberculosis, geographical and socioeconomic factors among patients examined at Mubende regional referral hospital, Uganda.

Variable	Label	Number ofpatients	TB statusn (%)
Patients	Sampled	344	74 (21)
HIV status	Negative	60	14 (23)
	Positive	82	46 (56)
	Unknown	202	14 (7)
Sample type	Sputum	294	65 (88)
	Lymph node aspirate	50	9 (12)
Sex	Male	207	50 (24)
	Female	138	24 (17)
Age	Youth (<20)	36	7 (19)
	Adult (20–45)	257	54 (21)
	Old (>45)	52	12 (23)
Marital status	Single	143	33 (23)
	Married	165	29 (17)
	Divorced	26	6 (23)
	Widowed	11	5 (45)
Household size	<6	129	10 (7.7)
	6–10	200	52 (26)
	>10	13	11 (84)
Ethnic group	Ganda	69	19 (27)
	Nyoro	76	22 (28)
	Batooro	23	2 (8)
	Bakiga	47	7 (15)
	Nyarwanda	27	4 (15)
	Basoga	13	1 (7.7)
	Banyankole	56	11 (19)
	Bafumbira	15	5 (33)
	Others*	18	2 (11)
**Geographic**	**Sub-county**		
Buwekula	Madudu	40	14 (35)
	Kasambya	26	7 (27)
	Town council	36	9 (25)
	Kiyuni	20	9 (45)
	Bagezza	70	17 (24)
	Butologo	17	2 (11)
Kassanda	Bukuya	27	1 (3)
	Myanzi	33	0
	Kiganda	19	1 (5)
	Kitenga	37	8 (22)
	Kasanda	19	1 (5)

### TB Prevalence and Associated Risk Factor

The mixed effects multivariable logistic model shows that an individual diagnosed with TB (based on culture) was more likely to be co-infected with HIV. Additionally, TB patients were more likely to come from a household size of between 6–10 people. Smoking and a history of previous TB treatment were also associated with TB prevalence. There was more variation in TB prevalence between sub-counties than within sub-counties (Table 2).

**Table 2:** Mixed effects logistic model showing the factors associated with prevalence of tuberculosis in Mubende district.

Variables	Level	Odd ratio(95% CI)	P-value
**HIV status**	Negative	1	–
	Positive	2.7(0.97–7.26)	0.050
	Unknown	0.15(0.54–0.46)	0.001
**Household size**	<6	1	–
	6–10	4.8(1.75–13.19)	0.002
	>10	0.0039(−)	0.986
**TB history**	Newly diagnosed	1	–
	Previously treated	3.3(1.32–8.31)	0.010
**Smoking status**	Non smoker	1	–
	Smoker	3.5(1.53–8.35)	0.003
**Medical related bills**	Consultation	1	–
	Drugs	5(1.87–16.63)	0.002
	Transport	13(4.87–36.53)	0.001

The random effect (sub-county) showed a variance of 1.12 (0.51–2.43).

### Economic Analysis

The economic aspects of tuberculosis were analyzed using a mixed effects multivariable linear regression model (Table 3). Fifty-eight percent (n = 199) of the interviewed patients could be categorized as living below the poverty line as they earned less than 2500 shillings ( = 1$) per day. Thirty-nine percent did not report any source of income while only eight percent could earn enough to spend a dollar per day, the majority of whom fell under the category of professionals. The model revealed that a female earned 20,000/ = (8$) per month less than their male counter parts. On the other hand the older patients (≥45 years) earned 25,000/ = (10$) more than the younger patients (≤20 years). HIV positive individuals earned 31,000/ = (12$) more than patients that were HIV negative. The results also showed that individuals who on average earned 58,793/ = ($22) and 110,905/ = ($42) spent most and least on leisure and health respectively. Patients were more likely to spend most on medicine and medical related transport bills respectively.

**Table 3:** Mixed linear regression model showing the relationship between monthly income in Uganda shillings and social and health factors among sampled patients with tuberculosis at Mubende regional referral hospital.

Variables	Level	Coefficient (95% CI)	P-value
**Sex**	Male	1	–
	Female	−20802 (−29151–12453)	0.050
**Age**	Young (<20)	1	–
	Adults (20–45)	10506 (−3186–24200)	0.133
	Old (>45)	25524 (8788–42260)	0.003
**Household size**	<6	1	
	6–10	7770 (−927–16468)	0.080
**HIV status**	Negative	1	–
	Positive	31859 (18793–44926)	0.001
	Unknown	891 (−10508–12290)	0.878
**Items with most expenditure**	Education	26119 (−8635–60873)	0.140
	Leisure	58793 (25194–92393)	0.001
**Items with least expenditure**	Education	−15898 (−39861–8065)	0.193
	Health	110905 (65581–156229	0.000

The random effect (tribe) showed a variance of 8296 shillings (3654–18835). At the time of study 1USD was equivalent to 2500 Uganda shillings.

### Identification of Isolates

Deletion analysis was performed on 74 isolates of MTC. For an isolate to be classified as *M. tuberculosis*, all four genomic regions of difference examined should be present (RD1, RD4, RD9 and RD12). A total of 72 isolates were designated as *M. tuberculosis*. The remaining two isolates did not yield any bands, the same isolates did not yield results from MIRU-VNTR and spoligotyping.

Seventy-two isolates were submitted to Genoscreen® for spoligotyping and MIRU/VNTR. Five out of these isolates gave multiple amplifications on MIRU-VNTR and weak hybridization signals on spoligotyping and were excluded from further analysis.

### Spoligotyping

**Figure 1:**
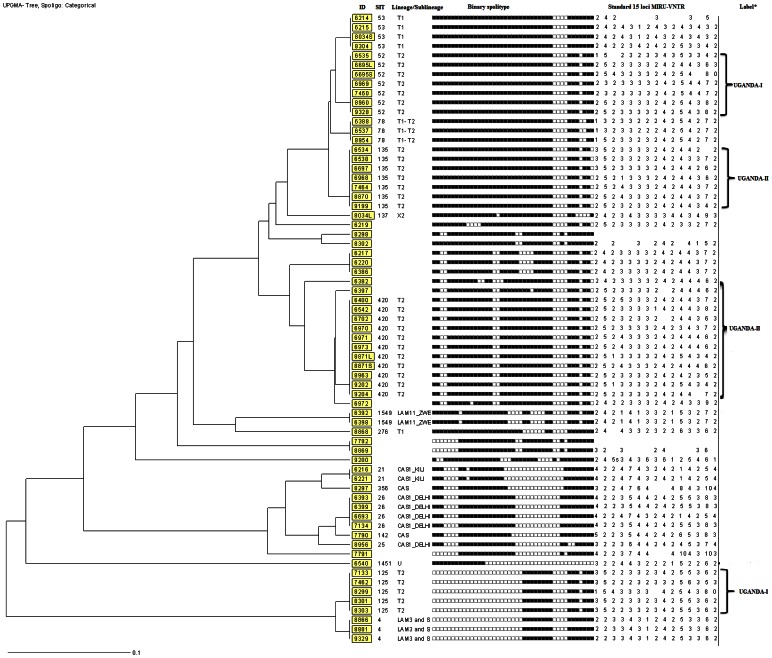
UPGMA phylogenic tree (MIRU-VNTR *plus*) based on spoligotype analysis and MIRU-VNTR of *Mycobacterium tuberculosis* isolates from patients at the Mubende regional referral hospital, Uganda. Standard International types (SIT), lineages and MIRU-VNTR data are presented.

**Figure 2:**
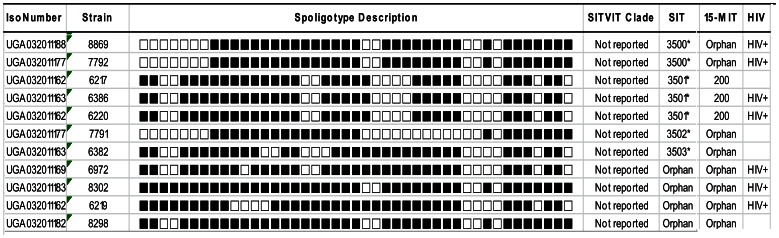
Novel spoligopatterns recently assigned SITs in the SITVITWEB database at the WHO supranational laboratory, Institute Pasteur de Guadeloupe [38].

Spoligotyping results revealed 23 different spoligo-patterns with an overall cluster rate of 62% and a discriminatory power of 93.7% (Figure 1). A total of 52 isolates (78%) were grouped in 12 clusters ranging from 2 to 11 isolates (Figure 1 and Table 4). Fifteen isolates did not cluster, nine of which were novel and thus did not exist in any of the data bases used for this study. Isolates that did not exist in the SITVITWEB database were submitted to the management of the database and were assigned new Shared international types (Figure 2). SIT420 was the most prevalent with a cluster rate of 14.8%, and mostly recovered from rural residents (Figure 1 and Table 4). SIT52, SIT135 and SIT125 also had a relatively high cluster rate of between 6 and 9%. These too were predominantly isolated from rural dwellers. Using the MIRU-VNTR *plus* online resource the isolates were further differentiated into six lineages namely T1, T2, CAS, X2 and LAM (Figure 1). Previous studies have classified *M. tuberculosis* isolates that belong to the T2 lineage as Uganda-I and Uganda-II, based on the lack of hybridization to either spacer 40 or both 40 and 43 respectively [24], [26], [27].

**Table 4:** The estimated transmission rate (cluster rate) of *M. tuberculosis* genotypes recovered from patients at Mubende regional referral hospital, Uganda Mubende district.

Lineage	SIT	Sub-type	Source (%)Rural Urban		Number ofclusters	Clustersize (x)	Identical onboth tools** (y)	Cluster rate (%) RCR ACR	
T1	53		25	75	1	4	2	5.4	2.7
T2	52	Uganda-I	86	14	1	7	4	9.5	5.4
T2	125	Uganda-I	100	0	1	5	2	6.7	2.7
T2	135	Uganda-II	58	42	1	7	2	9.5	2.7
T2	420	Uganda-II	64	36	1	11	0	14.8	–
T1–T2	78		0	100	1	3	2	4	2.7
LAM11-ZWE	1549		100	0	1	2	2	2.7	2.7
CAS1-KILI	21		50	50	1	2	2	–	2.7
CAS1-DEHLI	26		0	100	1	4	2	5.4	2.7
LAM3 and S	4		50	50	1	3	3	–	4
Unknown	3500*		0	100	1	2	0	2.7	–
	3501*		0	100	1	3	3		4
**Total**					**12**				

The total number of isolates is 67 (N).

SIT is shared international type, RCR (relative cluster rate) = (x)/N, ACR (Absolute cluster rate) = (y)/N, Urban = (Bagezza and Madudu subcounty), Rural = (the rest of the sub counties), *Recently assigned SIT, **Spoligotyping and MIRU-VNTR.

### MIRU-VNTR

**Figure 3:**
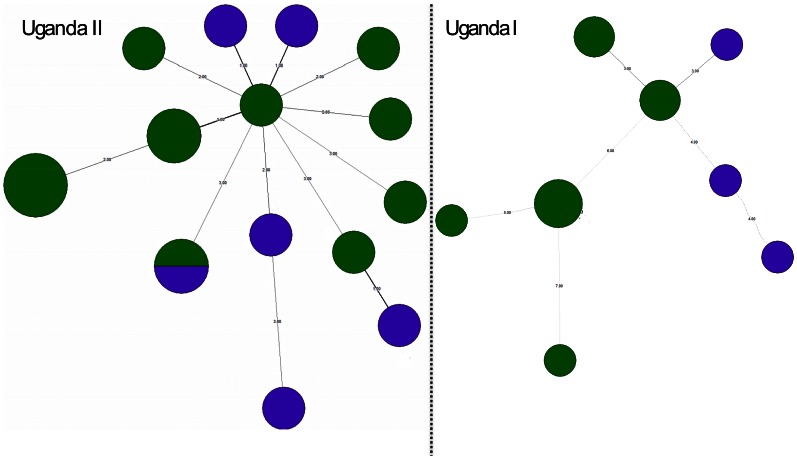
An unrooted minimum spanning tree (MST) showing the molecular relationships of clonal variants of Uganda-I and Uganda-II based on 15 standard MIRU-VNTR loci (Bionumerics 6.1). The purple and green colors represents isolates from urban (Mubende town council and Bagezza) and rural settings (the rest of the sub counties) respectively. In this figure the length and thickness (edged) and steps represent the genetic relatedness between any two connected clonal variants. The size of the nodes (circle) represents the number of isolates of the clonal variants of the T2-Uganda genotypes.

When the isolates were analyzed with MIRU-VNTR *plus*, the panel of 15 loci gave an overall discriminatory power of 97.1%. MIRU-VNTR data are presented in Figure 1. Within cluster analysis using an unrooted minimum spanning tree (MST) of the two predominant clusters (Uganda-I and Uganda-II), revealed that Uganda-II was more homogeneous given the fewer steps and thicker lines between the clonal variants as well as larger nodes. This high genetic relatedness is also shown by the narrow range in the *K* value (80%–93.3%) (Table 5 and Figure 3). On the other hand, Uganda-I was quite heterogeneous given the thin, long lines and more steps between clonal variants as size of circles which correspond to lower genetic relatedness between the clonal variants. This is also emphasized by the wide *K* range (53.3–80%).

**Table 5:** The percentage genetic relatedness between two clonal variants.

Number of steps n^ith^	Genetic relatedness *K* (%)
1	93.3
2	86.6
3	80
4	73.3
5	66.6
6	60
7	53.3

Minimum spanning tree calculations are based on the formula in materials and methods and the steps in Figure 3.

### Drug Resistance Patterns among the Clinical Isolates

Among the 67 isolates for which the drug susceptibility test was done, 65% was detected in newly diagnosed and 33% in previously treated patients (Table 6). Thirteen isolates were resistance to at least one drug, and the majority of the drug resistant isolates were recovered from the patients that had been previously treated for tuberculosis. Isoniazid resistance was found in SIT4, SIT52 and SIT420, while rifampicin resistance was found in SIT52 and SIT142. Resistance against streptomycin was found in SIT26, SIT53, SIT142, SIT1451 and SIT1549, while resistance to ethambutol was detected in SIT52 and SIT420. Two individuals from Kassanda harbored isolates that were resistant to both rifampicin and isoniazid hence classified as MDR-TB (Table 6). One of them presented as a newly diagnosed TB case with an unknown HIV status, while the other was HIV positive with a previously treated TB infection. Both were infected with SIT52. The isolate from the HIV positive MDR-TB patient was also resistant to all the other drugs tested in this study.

**Table 6:** Anti-tuberculosis drug resistance in *M. tuberculosis* isolates from new and previously treated patients at Mubende regional referral hospital, Uganda.

Resistance pattern	Newly diagnosed N (%)	Previously treated N (%)	Total N (%)
Total tested	45	22	67
Susceptible	36 (80)	18 (82)	54 (81)
**Resistance to one drug**			9 (13)
R	0	2	2
I	1	2	3
E	1	1	2
S	2	0	2
**Resistance to>one drug**			4 (6)
I+R*	1	0	1
I+R+E+S*	0	1	1
I+R+E*	1	0	1
I+S	1	0	1

R = rifampicin, I = isoniazid, E = ethambutol, S = Streptomycin, *MDR = multi drug resistance and resistance to Isoniazid and rifampicin.

## Discussion

### TB Prevalence and Risk Factor Analysis

This study documented the prevalence of tuberculosis among individuals who presented with a two week’s persistent cough and/or cervical lymphadenitis at Mubende referral hospital. *M. tuberculosis* was isolated from 21% of the patients, 62% of whom were also HIV positive. This co-infection is also shown by the mixed effects multivariable logistic regression model which reveals that a TB infected individual was more likely to either be a case of TB relapse or HIV/TB co-infection. HIV/TB co-infection has been documented over the years with various interpretations to the relationship [4], [5]. The incidence and case fatality rates are higher in HIV infected patients given their immune deficiency [4], [5]. A TB patient was more likely to be a member of a household of between 6 to 10 individuals. Tuberculosis is an air borne disease, therefore overcrowding at household level can be a risk [22]. In South Africa however, findings showed that overcrowding was more of a risk for children [16]. Studies recently carried out in Taiwan and Nigeria highlighted the role of cigarette smoking in the TB pathogenesis and case fatality rates [8], [9]. The findings in this study seem to concur with these studies, because a TB positive patient was also more likely to be a cigarette smoker.

### Economic Analysis

The economic impact of TB is enormous in sub Saharan Africa [18]. TB positive patients in this study were likely to spend most on medicine and medically related transport bills. This phenomenon has also been reported in Zambia where TB patients spend more on therapy and transport per month [19]. In Mubende, high transport costs which result from poor transport infrastructure [15] could have a negative effect on adherence to treatment which might inherently affect the bacterial susceptibility to current therapeutics and hence promote drug resistance. TB/HIV is indeed affecting the breadwinners in this community. Not only is it affecting the would-be productive age group, but also the economically sound individuals. This is supported by the regression model of monthly income of patients which revealed that HIV infected patients earned more than HIV negative patients. This could imply that once incapacitated, the families dependent on these individuals are left helpless, thus the cycle of poverty described in developing countries [16]–[19].

The triple burden effect of housework, childcare and employment allows women in developing countries very little time to access health centers for TB treatment [16]–[19]. In Mubende however, this disadvantageous status could be exacerbated by the disparity in monthly income, where women earned much less than their male counterparts. This disparity can be traced back to pastoral traditions. Women are rarely assigned major economic responsibilities, which not only limits their ability to earn but also their health seeking behavior [10].

### Molecular Epidemiology

The results demonstrated a genetic heterogeneity of *M. tuberculosis* isolates from Mubende. Isolates from the T2 lineage were the most predominant with a relative cluster rate of 40.5% which also indicates recent transmission. This finding supports the suggestion that this lineage is well established in Uganda [25]–[28], [42], in contrast to other East African countries where the incidence of T2 is much lower [20]. Previous studies have classified *M. tuberculosis* isolates that belong to the T2 lineage as Uganda-I and Uganda-II [24]–[26].

SIT125 clustered in a small but peculiar group which to date had only been isolated in Kampala [25]. In Mubende, it was however almost exclusively isolated from residents of Madudu and Butologo. This could be due to the fact that many of the urban dwellers in Kampala hail from rural areas, and when infected with chronically debilitating diseases like HIV and TB, it is common that they return to their ancestral homes in the terminal stages of disease. This exodus may be the source of SIT125 in these rural sub-counties of the Mubende district. This geographical exclusivity seems to fit the epidemiological data which indicates a higher variation between than within sub-counties. Similarly, SIT26 was exclusively recovered from urban dwellers, possibly an indication of differences in urban and rural TB dynamics. This seems to be supported by the fact that SIT26 is a central Asian TB strain which are prevalent on the Indian sub-continent [20] and that hosts from this area mostly settle in urban areas of Africa. SIT53, SIT1451 and SIT137 are widely prevalent in the former Soviet Union territories, United States and United Kingdom respectively [20], [21]. Given that these too were predominantly isolated from urban dwellers, this could reflect the connectivity and mobility among individuals in urban settings. The study has led to the identification of four new Shared international types in the SITVITWEB data base. It is noteworthy that the majority of these new SITs were recovered from HIV infected patients, supporting the theory that pathogen evolution is most rampant among HIV infected patients [43].

Spoligotyping reflects mutations that occur over a long period of time while MIRU-VNTR with its high definition reflects the subtle mutations that have occurred over a relatively shorter periods [21]. Uganda-II was more homogeneous than Uganda-I which could either imply that there are fewer exogenous clonal variants of Uganda-II introduced or it has a low mutation propensity. This difference in polymorphism between the two predominant clusters could be a reflection of the dynamics occurring at the individual level [30], [43]. The majority of the individuals from which Uganda-I was isolated were rural residents, something that could support the hypothesis of a long delay between infection and diagnosis of TB [10], given the long distance patients in rural areas would have to travel to access the TB health care services which are usually found in urban areas of Mubende district [15]. This delay could then give the infecting *M. tuberculosis* population enough time to multiply to levels that can trigger microevolutions [43]. The other possibility could be exogenous additions of clonal variants of Uganda-I from Kampala where it is reported to be predominant [25], [42].

### Drug Resistance Patterns among the Clinical Isolates

The majority of the isolates recovered were susceptible to the currently available first line of treatment against tuberculosis. However, 19% of the isolates were resistant to at least one drug. There was no difference in the proportions of resistant isolates recovered from newly diagnosed and previously treated TB cases, a finding that contradicts previous reports from Uganda [12], [14]. This is most likely to be because of the few numbers of drug resistant isolates recovered which could not statistically support the detection of differences between and within groups. Two MDR-TB isolates were recovered, one from a newly diagnosed and one from a previously treated TB case. The vast majority of resistance was observed in local genotypes which seems to supports reports from Turkey suggesting that the bulk of resistant strains are usually endemic to local populations. This is also most likely to be a challenge to the current DOTS control strategy in Mubende, since the most prevalent genotypes also exhibits the highest level of resistance to drugs used in the first line treatment of TB.

### Conclusions

The study detected *M. tuberculosis* from 21% of examined patients presenting with TB like symptoms in the Mubende district of Uganda, 62% of whom were also HIV positive. The majority of isolates were sensitive to the first line treatment drugs, but resistance was detected in 19% of the isolates. Molecular analysis demonstrated a heterogeneous pool of *M. tuberculosis* strains circulating in the area. HIV/TB co-infections, high medical related transport bills and drug resistance could undermine the usefulness of the current TB strategic interventions.

## Supporting Information

Questionnaire S1 The epidemiology of tuberculous and non-tuberculous mycobacteria in Mubende district, Uganda.
(PDF)
