# Supplementary material for: Molecular Epidemiology, Drug Susceptibility and Economic Aspects of Tuberculosis in Mubende District, Uganda
Source: PLoS One. 2013 May 31;8(5):e64745. doi: 10.1371/journal.pone.0064745 (PMC3669366; doi:10.1371/journal.pone.0064745)
Supplement: Questionnaire S1 — The epidemiology of tuberculous and non-tuberculous mycobacteria in Mubende district, Uganda. (PDF) [file pone.0064745.s001.pdf]

Makerere University
"we build for the future"

Questionnaire
For
The epidemiology of tuberculous and non-tuberculous mycobacteria in Mubende district, Uganda

Author: Adrian Muwonge **Sero status…………………..**

Case number………………….. **Date…………………………**

Do you consent to participating in this study Yes...…………………… ☐ NO……………………..… ☐

	**BIODATA**	
Sex……………………………	Age………………………	Wieght………………………
Height…………………………	Tribe………………………	Occupation…………………...
Sub county……………,,,,,,,….	Ward………………………..	
Family size…………………		
	**Risk Factors**	
Previous respiratory infection<br>Yes……………………… ☐<br>NO……………………… ☐	Previous T.B infection<br>Yes...…………………… ☐<br>NO……………………… ☐	Previous lymphadenitis<br>Yes...…………………… ☐<br>NO……………………… ☐
Do you smoke<br>Yes……………………… ☐<br>NO……………………… ☐	Drink alcohol<br>Yes...…………………… ☐<br>NO……………………… ☐	Eat pork<br>Yes……………………… ☐<br>NO……………………… ☐
Roasted meat<br>Yes……………………… ☐<br>NO……………………… ☐	Raw milk<br>Yes………..……………… ☐<br>NO………..……………… ☐	Use pig manure<br>Yes……………………… ☐<br>NO……………………… ☐
Do you rear pigs<br>Yes……………………… ☐<br>NO……………………… ☐	If yes,<br>Subsistence……………… ☐<br>commercial……………… ☐	What is your water source<br>…………… ……………..<br>…………… ……………..<br>……………
Do you boil your drinking water<br>Yes……………………… ☐<br>NO……………………… ☐	Have you ever taken unboiled water<br>Yes……………………… ☐<br>NO……………………… ☐	If no<br>Is there any other mode of water treatment used?<br>………………………………..
Do you grow your own food or buy it from market<br>Yes……………………… ☐<br>NO……………………… ☐	How often do you buy food stuffs from the Market<br>……………………………<br>………………………………	Do you keep livestock<br>Yes……………………… ☐<br>NO……………………… ☐<br>If yes which<br>Cattle….……………………… ☐<br>Pigs………………………… ☐<br>Shoats……………………… ☐<br>Poultry…..………………… ☐
	**Therapeutics (Medical record)**	
What TB medication previously used<br>……………………………<br>……………………………<br>……………………………	Currently used<br>……………………………..<br>……………………………..<br>……………………………..<br>……………………………	For how long<br>……………………………...<br>………………………………<br>……………………………..<br>……………………………

last deworming<br>within last three months....☐<br>within last one year……...☐<br>Never ……………………☐		

	**Social Networks/contacts**	
Marital status<br>Single……………………☐<br>Married……………………☐<br>Widowed…………………☐	How often do you come to Hospital in a year<br><br>……………………………<br>……………………………	Do you go to places of worship<br>Yes………………………☐<br>NO………………………☐
IF yes which one and how often?<br>……………………………<br>……………………………	Have you been to school/s (parents day, visiting day etc) in the last 8 months.<br>Yes………………………☐<br>NO………………………☐	Have you been to any social gathering in the last 8 months (wedding, last funeral rights, soccer game, bar.) etc<br>Yes………………………☐<br>NO………………………☐
Have you travelled away from your sub-county in the last 8 months?<br>Yes………………………☐<br>NO………………………☐	If yes which places<br>Mubende TC………………☐<br>Mityana…….……………...….☐<br>Myanzi……....……………☐<br>Kagadi……....……………☐<br>Kampala…………………☐<br>Others………………………	

	**Economic Aspects**	
How much on average do you earn per months………………………	What do you spend on most.<br>Education………………..☐<br>Domestic expenses………☐<br>Health related…………...☐<br>Leisure related…………☐<br>Others……………………☐	If health. What do u spend on most?<br>Drugs……………………☐<br>Transport………………..☐<br>Consultancy……………..☐<br>Nutrition…………………☐
If leisure, what do u spend on most<br>Alkohol…………………☐<br>Disco……………………☐<br>Travel……………………☐<br>Gambling………………...☐	What do u spend on least<br>Education………………..☐<br>Domestic………………...☐<br>Health related……………☐<br>Leisure…………………...☐<br>Others …………………☐	
